# The hedgehog pathway in triple‐negative breast cancer

**DOI:** 10.1002/cam4.833

**Published:** 2016-08-18

**Authors:** Joyce G. Habib, Joyce A. O'Shaughnessy

**Affiliations:** ^1^ Baylor Charles A. Sammons Cancer CenterDallasTexas; ^2^ Texas OncologyDallasTexas

**Keywords:** Breast cancer stem cells, EMT, hedgehog, invasion, metastasis, RTK, TGF‐*β*, TNBC

## Abstract

Treatment of triple‐negative breast cancer (TNBC) remains challenging due to the underlying heterogeneity of this disease coupled with the lack of predictive biomarkers and effective targeted therapies. Intratumoral heterogeneity, particularly enrichment for breast cancer stem cell‐like subpopulations, has emerged as a leading hypothesis for systemic therapy resistance and clinically aggressive course of poor prognosis TNBC. A growing body of literature supports the role of the stem cell renewal Hedgehog (Hh) pathway in breast cancer. Emerging preclinical data also implicate Hh signaling in TNBC pathogenesis. Herein, we review the evidence for a pathophysiologic role of Hh signaling in TNBC and explore mechanisms of crosstalk between the Hh pathway and other key signaling networks as well as their potential implications for Hh‐targeted interventions in TNBC.

## Introduction

Triple‐negative breast cancer (TNBC), defined immunohistologically by the absence of estrogen (ER), progesterone (PR), and HER2 receptors, is a heterogeneous entity comprising subtypes with distinct clinical and molecular features 1, 2, 3, 4. Despite some variability in clinical course, the prognosis of TNBC patients is poorer overall than those with stage‐equivalent non‐TNBC 5, 6, 7.

The advent of high‐throughput sequencing technologies and bioinformatics has improved our understanding of the molecular diversity of TNBC and opened new avenues for potential targeted therapeutic strategies 2, 3, 8. Differential response to neoadjuvant chemotherapy among molecular subtypes of TNBC has been reported 9; yet, prospective validation of molecular subtyping as a predictive tool in TNBC is currently lacking. In addition, targeted agents investigated by our group and others have not yielded convincing activity in unselected patients, highlighting an unmet need for novel therapeutic strategies in TNBC (reviewed in 10).

The identification of breast cancer cells with self‐renewal and multilineage differentiation capacity led to the hypothesis that tumor growth and heterogeneity are driven by subpopulations of breast cancer stem cells (BCSCs) 11, 12. Cellular markers, CD44^+^/CD24^−/low^ phenotype 11 and aldehyde dehydrogenase 1 (ALDH1) activity 12 along with functional assays, such as anchorage‐independent growth and mammosphere formation, are commonly used to isolate tumor subpopulations enriched for BCSCs 13, 14. Notwithstanding, the exact molecular features of this putative tumor cell subset currently remain undefined.

Because of their intrinsic chemoresistance 15, 16 and propensity for invasion and metastasis 17, 18, it has been suggested that enrichment for BCSCs is associated with aggressive tumor biology and poor clinical outcome 12, 19, 20, 21. A corollary to this premise is that enrichment for BCSC subpopulations in claudin‐low 22, metaplastic 23, and some basal‐like TNBC 24 may partly explain their aggressive clinical course and poor prognosis 25, 26. In this respect, enrichment for BCSC phenotype and genetic signature in TNBC was shown to negatively correlate with pathologic complete response 26 and has been associated with higher incidence of recurrence and metastasis 27.

Deregulation of receptor tyrosine kinase (RTK), TGF‐*β* signaling and embryonic pathways, Wnt, Hedgehog (Hh), and Notch have been recognized as key signaling events driving tumorigenesis in BCSCs 28, 29, 30, 31, 32. The Hh pathway plays a key role in embryonic development and regulates stem cell renewal and tissue homeostasis 33, 34, 35, 36. Its role as an oncogenic pathway in basal cell carcinoma and medulloblastoma is well established 37, 38. A growing body of literature substantiates the role of deregulated Hh signaling in breast cancer 39, 40, 41 with emerging data also highlighting its pivotal contribution to TNBC pathophysiology 42, 43, 44, 45, 46.

Herein, we review the evidence supporting a pathogenic role for Hh signaling in TNBC. We also discuss mechanisms of Hh pathway activation highlighting the critical contribution of extrinsic mediators and other key oncogenic pathways to deregulated Hh signaling in TNBC. Insight into these mechanisms and their potential implication in bypass signaling promoting resistance to Hh inhibitors is crucial for the design of effective Hh‐ and BCSC‐targeted therapeutic strategies in TNBC.

## Hedgehog Signaling and Regulation

### The hedgehog signaling cascade

**Figure 1:**
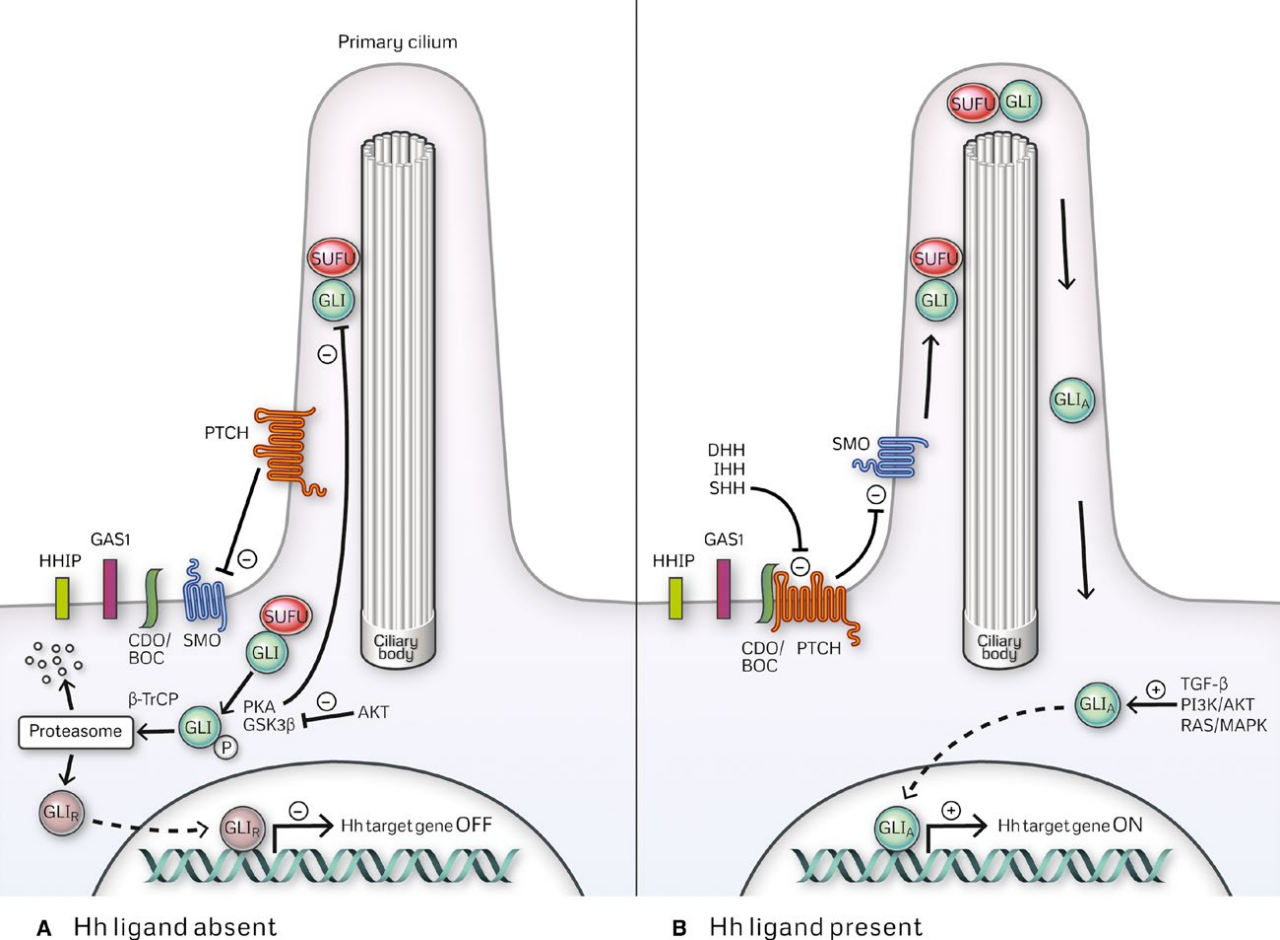
The Hedgehog signaling cascade. (A) In the absence of its ligand, the Hh receptor PTCH localizes to the primary cilium where it interferes with SMO ciliary trafficking and activation. GLI proteins are sequestered in the cytoplasm by SUFU where they undergo phosphorylation followed by either degradation or processing into repressor forms (GLI_R_). Both GLI2 and GLI3 undergo proteolytic modification into repressor forms GLI2_R_ and GLI3_R_, whereas GLI1, lacking a repressor domain does not. (B) Upon Hh ligand binding, PTCH suppression of SMO is relieved resulting in SMO ciliary translocation and activation. Cell surface receptors regulating Hh ligand‐PTCH interaction include positive regulators CDO* (cell adhesion molecule‐related/downregulated by oncogenes), BOC* (brother of Cdo), and GAS1* (growth arrest‐specific gene 1) and negative regulator HHIP (Hh‐interacting protein). Activated SMO promotes trafficking of SUFU‐GLI complexes to the distal cilium followed by dissociation of GLI proteins from SUFU. Activation of GLI1 and inhibition of GLI2 and GLI3 proteolytic processing occur leading to formation of full‐length GLI transcription factors in their activator form (GLI_A_). Nuclear translocation of GLI_A_ ensues and leads to upregulation of Hh target genes. Functional redundancy exists between GLI1 and GLI2; both GLI1 and GLI2 regulate the expression of overlapping target genes and GLI2 also upregulates GLI1 expression. GLI‐mediated transcriptional output is also influenced by the context‐dependent activator/repressor functions of GLI. Different combinations of activator and repressor forms of GLI regulate the expression of either distinct or partially overlapping sets of genes, ultimately leading to diverse cellular responses. *While the role of BOC, GAS1, and CDO has been described in non‐small‐cell lung cancer, pancreatic, and prostate cancer, a direct connection to Hh signaling in TNBC, specifically, has not been reported.

The Hh pathway plays an essential role in embryonic patterning and is involved in stem cell renewal, tissue regeneration, and repair 33, 34, 35, 36. It involves a signaling cascade mediated by three secreted ligands—Sonic Hedgehog (SHH), Indian Hedgehog (IHH), and Desert Hedgehog (DHH)—and transmembrane receptor and co‐receptor Patched (PTCH) and SMO, respectively (Fig. 1). Proper Hh signaling is also dependent on the presence of an intact primary cilium, a microtubule‐containing organelle projecting from the cell surface; its disruption abrogates the signaling cascade (reviewed in 47).

Three glioma‐associated oncogene (GLI) transcription factors, GLI1, GLI2, and GLI3, are the effectors of Hh signaling and regulate the expression of the pathway target genes 48, 49. GLI2 and GLI3 contain both activation and repression domains and can function as full‐length transcriptional activators (GLI_A_) or as truncated repressors (GLI_R_), whereas GLI1, lacking a repression domain, exists only as a full‐length transcriptional activator (GLI_A_) 50.

GLI2_A_ and GLI3_R_ are considered to be the primary transcriptional activator and repressor of Hh signaling during embryogenesis, respectively; conversely, GLI1_A_ is dispensable for development 51, 52. GLI3_R_‐mediated suppression of Hh target gene transcription is a critical event for mammary bud formation 53. Repression of the Hh pathway, as evidenced by the lack of transcriptional reporters of positive Hh signaling, is also required for proper embryonic and post‐natal mammary gland development 53. Therefore, during normal development, GLI1 expression is absent and GLI2 activity antagonized by GLI3_R_ in the mammary gland parenchyma 53.

In contrast, GLI1 and GLI2 are considered to be the main oncogenic effectors of Hh signaling, with lack of data supporting the contribution of GLI3 to breast carcinogenesis 39, 40, 42, 43, 44, 45, 54, 55, 56. To date, numerous GLI1 and GLI2 targets, involved in cell proliferation and survival, EMT, invasion, migration, angiogenesis, osteolytic metastases and drug resistance, have been identified (Table 1).

**Table 1:** Transcriptional targets of Hedgehog signaling

	Hedgehog signaling target genes
**Cell proliferation and survival**	CCND1 (cyclin D1)^a^
	BMI‐1 (BMI1 polycomb ringer finger oncogene)
	P63
	FOXM1 (forkhead box M1)^a^
	BCL‐2 (B‐cell CLL/Lymphoma 2)^a^
**EMT**	SNAI1 (snail family zinc finger 1)
	FOXM1Mechanism: FOXM1‐mediated upregulation of EMT transcription factor Slug by FOXM1 reported in TNBC
	FOXC2^a^ (forkhead box C2 (MFH‐1, mesenchyme forkhead 1)Mechanism not well understood in TNBC. Downregulation of the E‐cadherin stabilizing protein p‐120 catenin by FOXC2 has been described in non‐small‐cell lung cancer
**Invasion, migration, angiogenesis**	VEGF (vascular endothelial growth factor A)
	NRP2 (neuropilin 2)
	CYR61 (cysteine‐rich, angiogenic inducer, 61)
	MMP (matrix metalloproteinase) 2, MMP 9, MMP 11
	FOXM1 via regulating the expression of extracellular matrix degrading factors uPA (urokinase plasminogen activator), uPAR (urokinase plasminogen activator receptor), MMP2, MMP 9 along with VEGF
	CXCR4 (chemokine receptor 4)
**Osteolytic metastases**	PTH‐rP (parathyroid hormone‐like hormone)
	OPN (SSP1, secreted phosphoprotein 1)
**Chemotherapy resistance**	ABCB1 (ATP‐binding cassette, subfamily B, member 1)
	ABCG2 (ATP‐binding cassette, subfamily G, member 2)Anthracycline and taxane resistance.Mechanism: active drug efflux
	FOXM1:Anthracycline and cisplatin resistance. Mechanism: induction of double‐stranded DNA repair gene expressionTaxane resistanceMechanism: upregulation of the protein stathmin leading to microtubule disruption and interfering with paclitaxel microtubule binding
	BMI‐1Resistance to five fluorouracil (5‐FU)Mechanism: BMI‐mediated inhibition of mitochondrial apoptotic pathways induced by 5‐FU

a Established transcriptional targets in non‐mammary cells/tumors.

FOXC2 is the target Hh signaling and other developmental pathways.

### Regulation of hedgehog signaling

The GLI‐driven transcriptional program is modulated by intrinsic and extrinsic regulation of GLI activity through post‐translational modification, such as phosphorylation, ubiquitin‐mediated degradation, acetylation, as well as regulation of nucleocytoplasmic shuttling (reviewed in 57). GLI phosphorylation by kinases—Protein kinase A (PKA), glycogen synthase kinase‐3beta (GSK3*β*), and casein kinase 1 (CK1)—creates binding sites for adaptor protein Beta‐transducin repeat containing E3 ubiquitin protein ligase (*β*‐TrCP), and marks GLI proteins for ubiquitin‐mediated proteasomal degradation or processing into repressor forms 58, 59. Another intrinsic regulatory mechanism involves GLI acetylation which interferes with the transcriptional activity of GLI1 and GLI2 by preventing their recruitment onto their target gene promoters 60, 61. Conversely, GLI1 and GLI2 deacetylation by Class I histone deacetylases (HDAC) enhances their transcriptional activity 60, 61.

Extrinsic regulation of Hh signaling by oncogenic pathways, such as RAS/MAPK, PI3K, and TGF‐*β* signaling, occurs through modulation of the expression or activity of Hh pathway components, predominantly GLI proteins. For instance, oncogenic and nonmutated aberrant RAS signaling enhance GLI1 function in pancreatic, gastric, and breast cancer through various mechanisms, including potentiation of its transcriptional activity and nuclear translocation, inhibition of its cytoplasmic sequestration by SUFU and proteasome‐mediated degradation 43, 62, 63, 64.

PI3K signaling also interferes with proteasomal GLI degradation by inhibiting PKA and GSK3*β*‐activity 65 and increased GLI1 expression in response to activated PI3K signaling has been shown to confer tamoxifen resistance in ER‐positive breast cancer 39. In addition, upregulation of *GLI1* and *SHH* expression mediated by NF‐*κ*B signaling has been reported in breast cancer 45, 66. Likewise, increased GLI2 expression in response to TGF‐*β*/SMAD signaling occurs through transcriptional regulation and is independent of downstream RAS/MAPK or PI3K/AKT signaling 67.

## Canonical and Noncanonical Hedgehog Signaling

Canonical Hh signaling has been defined as Hh ligand/receptor‐induced signaling leading to GLI activation. No consensus exists, however, on the definition of noncanonical Hh signaling. Cellular responses to Hh ligand, mediated by either PTCH (type I) or SMO (type II) independent of GLI, are classically considered as noncanonical signaling mechanisms 68. In addition, some have also included in this definition, mechanisms leading to GLI activation independent of Hh ligand‐mediated signaling 69. The definition we have adopted in this review comprises only type I or type II signaling, and we have referred to ligand/receptor‐independent GLI activation as canonical ligand‐independent signaling.

### Canonical Hedgehog signaling

**Figure 2:**
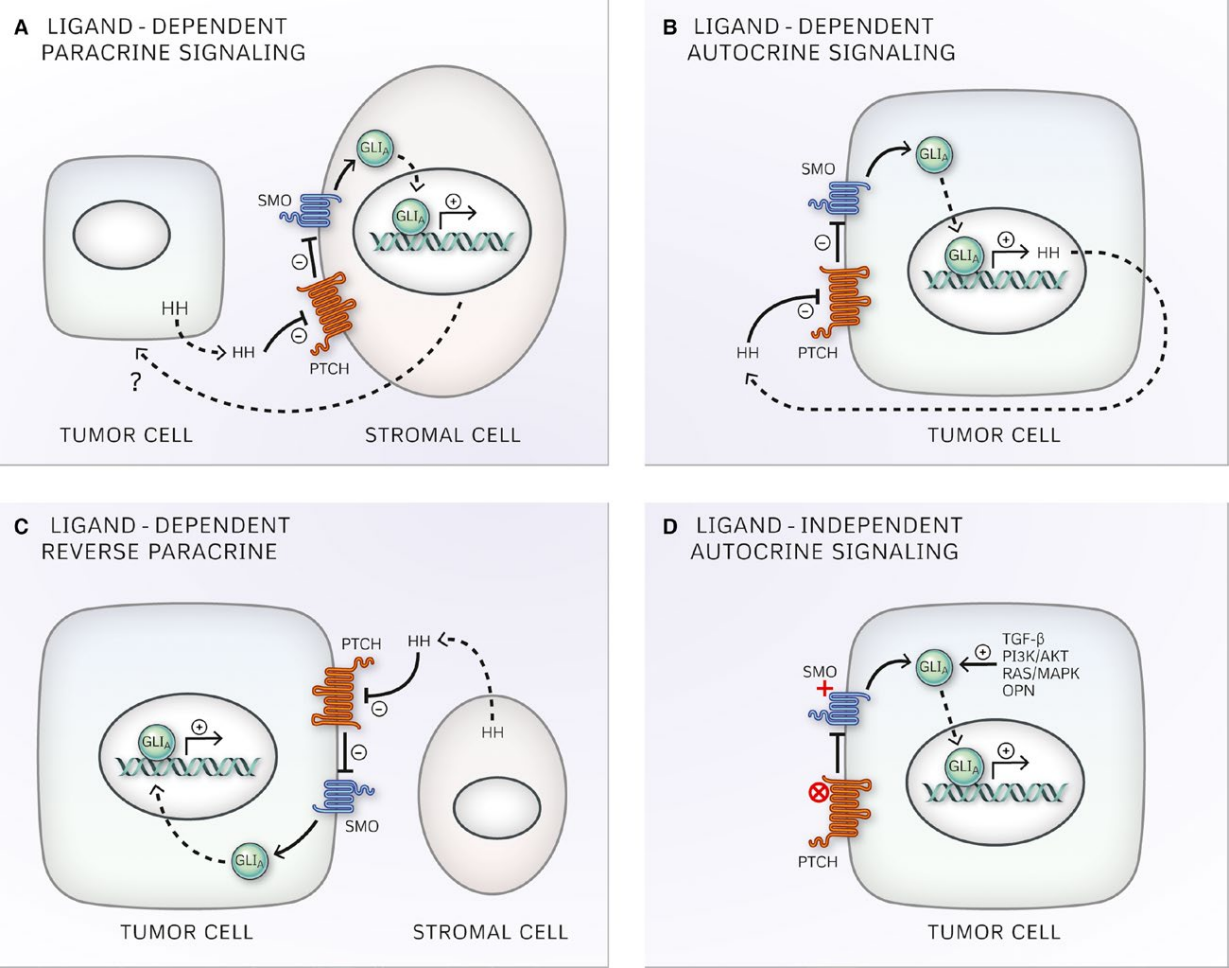
Mechanisms of canonical Hedgehog signaling in cancer: (A) Ligand‐dependent paracrine signaling, (B) Ligand‐dependent autocrine signaling, (C) Ligand‐dependent reverse paracrine signaling, (D) Ligand‐independent signaling.

Under this definition, mechanisms of canonical Hh signaling in carcinogenesis include ligand‐dependent signaling, further classified as paracrine, autocrine or reverse paracrine, and ligand‐independent signaling (Fig. 2).

Ligand‐dependent autocrine signaling occurs when cancer cells are activated by their secreted Hh ligand 70, 71. Ligand‐dependent paracrine signaling, on the other hand, occurs when tumor cell secretion of Hh ligand does not activate the pathway in the secreting cells. Instead, Hh ligand diffuses locally and activates canonical signaling in surrounding cancer and/or stromal cells 72, 73. Lastly, reverse paracrine signaling refers to activation of the pathway in cancer cells in response to stromal‐derived Hh ligand 74.

Ligand‐independent Hh signaling results in GLI activation independent of the presence of Hh ligand via mutational (loss of function PTCH mutations or activating SMO mutations) 37, 38, 75 or non‐mutational mechanisms (*GLI* amplification or extrinsic potentiation of GLI transcriptional activity) 62, 63, 64, 65, 76.

Current evidence suggests that Hh signaling in breast cancer overall, and TNBC specifically, is not mutation‐driven; infrequent somatic mutations in *SHH*,* PTCH*, and *GLI1* 77, 78, 79 have been reported; however, these findings have not been replicated in other studies 80, 81. Instead, three primary mechanisms of Hh signaling have been described so far in TNBC. These include ligand‐dependent paracrine and ligand‐dependent autocrine signaling and non‐mutational ligand‐independent signaling mediated by oncogenic pathways along with key transcription factors and extracellular matrix proteins as discussed below.

### Noncanonical Hedgehog signaling

Noncanonical Hh signaling mediated by PTCH (type I) has been shown to exert an antiapoptotic effect in nonmammary cells via disruption of proapoptotic DRAL‐caspase activity or inhibition of H‐Ras‐induced apoptosis by modulation of tumor suppressor protein Tid1 82, 83. Recent studies have also outlined the role of PTCH‐mediated noncanonical Hh signaling in regulating pubertal mammary ductal development 84, 85. In murine models, activation of c‐src, ER*α*, and downstream ERK1/2 signaling has been reported in mammary luminal epithelial cells in response to noncanonical PTCH‐dependent Hh pathway activation 84, 85, 86. Although the precise mechanism remains undefined, c‐src and ER*α* signaling likely exert a mitogenic effect on luminal progenitor cells leading to mammary ductal elongation from terminal end buds at puberty 84, 85. Despite evidence suggesting that similar noncanonical Hh signaling exists in non‐tumorigenic human mammary epithelial cells, its role in carcinogenesis is currently unknown 84, 86.

SMO‐dependent (type II) noncanonical Hh signaling promotes endothelial cell tubulogenesis, fibroblast and non‐mammary cancer cell migration by activation of small GTP‐ases Rho and Rac1 87, 88, 89. Nonetheless, at the present time, the existence and potential role of this signaling mechanism in breast carcinogenesis has not been investigated.

## Hedgehog Signaling in Triple‐Negative Breast Cancer

### Hh signaling and cancer stem cell reprogramming

Extensive preclinical data highlight the key contribution of Hh signaling in cancer stem cell reprogramming in TNBC 41, 42, 43, 44, 45, 55. A causal role for Hh signaling in regulating the stem cell factor BMI‐1 and conferring mammosphere‐forming ability *in vitro* and tumor initiation *in vivo* has been reported 43. Upregulation of *GLI1* expression is characteristically observed in claudin‐low breast cancer subtype mammospheres and tumors known to be highly enriched for BCSCs 43, 45. Contrariwise, suppression of mammosphere formation and reduced ALDH1 activity in TNBC is noted upon Hh pathway inhibition 45, 90.

The role of Hh signaling in EMT regulation has been described in several malignancies including ovarian 91, prostate 92, pancreatic 93, and lung cancer 94 with evidence also supporting its contribution to EMT in TNBC 45, 55, 95. Epithelial‐mesenchymal transition (EMT) is a crucial process for stemness acquisition in carcinogenesis 28, 96. It is a complex transdifferentiation process, orchestrated by several transcription factors (Zeb1/2, Snail1, Snail2 (Slug), Twist 1/2, Goosecoid, FOXM1, FOXC1, and FOXC2 among others) endowing breast tumor cells with enhanced self‐renewal, tumor initiating capacity, invasiveness and resistance to apoptosis 26, 28, 96, 97, 98. Hallmarks of EMT include loss of cell‐adhesion marker expression, such as E‐cadherin and claudins, and upregulation of mesenchymal markers such as vimentin and N‐cadherin 99, 100.

Expression of mesenchymal markers and EMT transcription factors in TNBC cells is under direct regulation of Hh effector GLI1; abrogation of Hh signaling leads to loss of the mesenchymal phenotype and restores E‐cadherin and epithelial marker keratin expression 55, 95. *In vivo* studies in murine mammary glands with constitutive Hh signaling reveal prominent ductal hyperplasia and dysplasia caused by expansion of cell populations exhibiting basal cytokeratin, P63, and progenitor marker expression along with loss of basolateral polarity reminiscent of EMT 101, 102. Moreover, the occurrence of ER‐negative basal‐like tumors characterized by loss of E‐cadherin along with expression of progenitor markers in murine models with conditional *GLI1* expression further corroborates the implication of the Hh pathway in EMT‐mediated tumorigenesis 103. Taken together, these data strongly suggest that establishment and maintenance of cancer stem cell phenotype in TNBC is orchestrated by Hh signaling.

### Hh signaling promotes tumor growth, invasion, metastasis, and drug resistance

Preclinical studies provide strong evidence that deregulated Hh signaling confers a more aggressive tumor phenotype in TNBC. Activation of the Hh pathway enhances the proliferation, invasion, and migration of TNBC cells 44, 46; conversely, its inhibition was shown to reduce their clonogenicity, self‐renewal capacity, and motility 42, 45, 46, 104. Increased angiogenesis 44 and expression of extracellular matrix degrading proteases 46, 105 are causative factors for the enhanced invasiveness and metastatic potential of TNBC cells observed upon Hh signaling activation.

An association between the Hh pathway and chemoresistance in TNBC has also been demonstrated. *In vitro* resistance to doxorubicin, paclitaxel, and cisplatin was shown to be mediated by GLI1 and was associated in TNBC cells with upregulation of multidrug resistance protein 1 (MDR1) and breast cancer resistance protein (BCRP), both transcriptional targets of Hh signaling (55 Supplemental data). Increased expression of multidrug resistance efflux transporters may, therefore, provide potential explanation for the observed Hh‐mediated survival and expansion of breast cancer cell subpopulations after taxane exposure 41.

*In vivo* data further lend support to the contribution of Hh signaling to aggressive tumor biology in TNBC, demonstrating enhanced local tumor invasion 56 with constitutive Hh signaling as well as increased incidence of visceral 44 and osteolytic metastases 54, 106. Despite suggestive preclinical data, studies attempting to explore the clinical significance of deregulated Hh signaling in breast cancer are limited. In addition, most reports seeking to establish correlations between Hh pathway activation and tumor characteristics or clinical outcome have included all breast cancer subtypes with a limited number looking at subtype‐specific associations. The ability to draw meaningful conclusions from these studies is further hampered by small sample size and the lack of uniform criteria or methodology used to define Hh signaling activation.

Keeping this in mind, a correlation between Hh signaling activation and high‐grade, larger (≥T2), highly proliferative tumors and TNBC histology has been reported 56. In TNBC, specifically, increased tumor GLI1 expression was found to correlate with higher tumor stage and node‐positive disease 107. More recently, Han et al.^90^ have shown that enrichment for Hh pathway‐associated genes is predictive of worse disease‐free survival in breast cancer patients. Although the latter study included the largest number of clinical samples to date, it did not assess the prognostic significance of the Hh genetic signature in specific breast cancer subtypes.

### Mechanisms of deregulated Hh signaling in TNBC: interplay of ligand‐independent and paracrine Hh signaling

#### Ligand‐independent Hedgehog signaling

**Figure 3:**
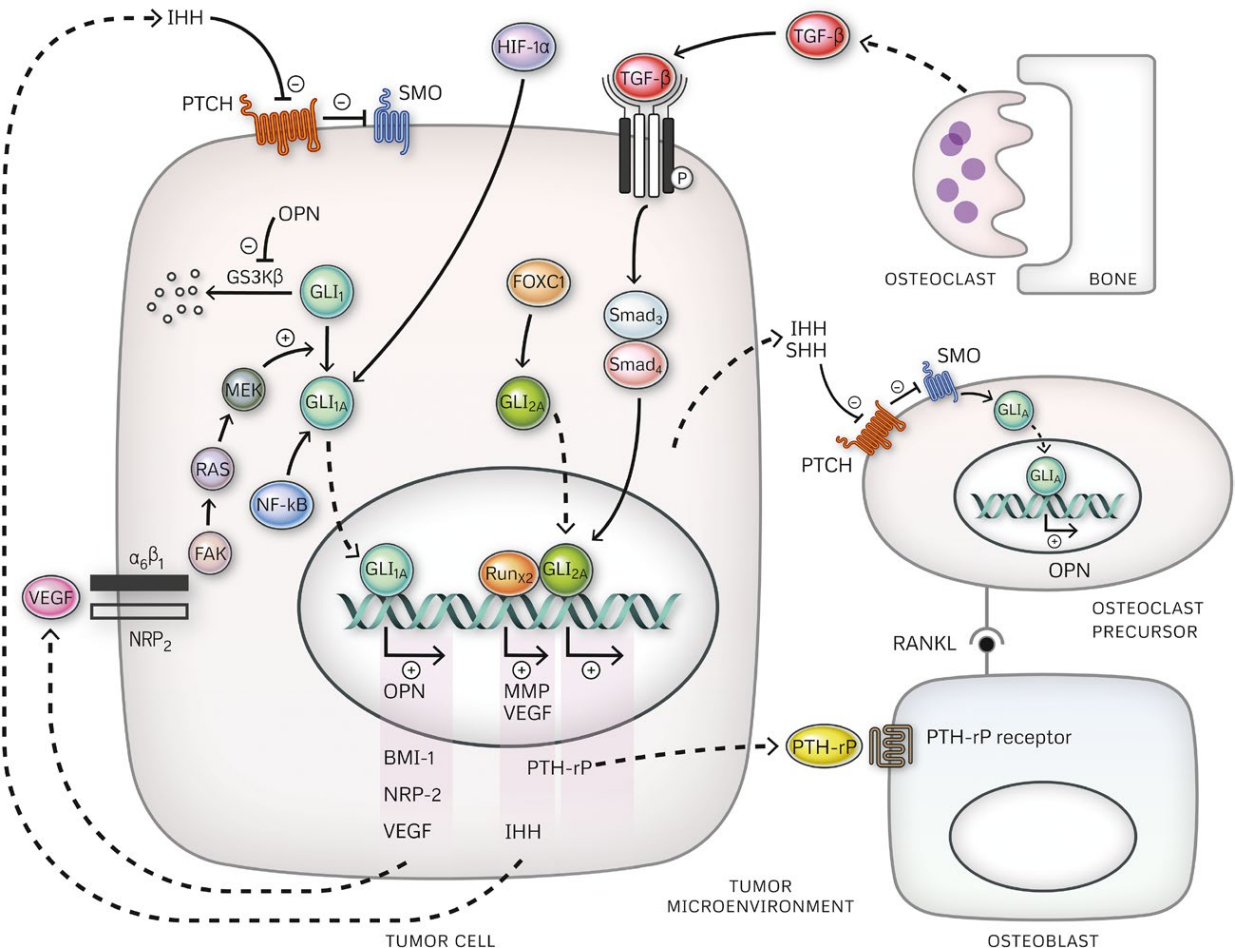
Hedgehog signaling activation in TNBC: interplay of ligand‐dependent and ligand‐independent mechanisms.

TGF‐*β*, RAS/MAPK signaling pathways, the extracellular matrix protein osteopontin (OPN), and transcription factors—NF‐kB, FOXC1, and Hypoxia‐induced factor (HIF)‐1*α*—contribute to deregulated Hh signaling, thereby, leading to acquisition of the mesenchymal phenotype, enhanced growth, and invasion in TNBC 43, 45, 54, 55, 90, 95 (Fig. 3). GLI transcription factors are, nonetheless, the targets of these distinct pathways; thus, representing an integration nexus for their signaling inputs. In this setting, Hh‐dependent gene transcription ensues independently of Hh ligand/receptor signaling activation 43, 54, 55.

Increased GLI2 expression and upregulation of its target gene PTH‐rP, in response to TGF‐*β*/SMAD signaling, plays a key role in the pathogenesis of osteolytic metastases in TNBC 54, 106. Osteomimetic Runx2 expression augments GLI2‐mediated PTH‐rP upregulation and by promoting *IHH* transcription*,* it further potentiates the vicious cycle of tumor growth and osteolysis mediated by TGF‐*β*/GLI2 108. VEGF Neuropilin 2 (NRP2) signaling in the triple‐negative BCSC compartment was shown to directly activate GLI1 through integrin‐mediated FAK signaling and downstream RAS/MAPK activation 43. GLI1‐mediated BCSC expansion was demonstrated in concert with increased expression of known GLI1 transcriptional targets, VEGF and NRP2 in tumor cells, suggesting the existence of a positive feedback loop promoting Hh signaling deregulation and driving tumorigenesis in TNBC 43. Hh pathway activation, underlying NF‐kB and FOXC1‐induced expansion of stem cell‐like tumor cells in TNBC, also occurs in a ligand‐independent fashion via direct potentiation of GLI1 expression and GLI2 activity, by NF‐kB and FOXC1, respectively 45, 90. Given these findings and taking into consideration the functional link between FOXC1 and NF‐kB in TNBC 109, the existence of a critical tumorigenic network involving Hh signaling, FOXC1, and NF‐kB cannot be excluded.

The tumor microenvironment is a key contributor to tumor growth and progression, and the importance of stromal signaling in regulating epithelial‐mesenchymal plasticity in breast cancer cells is increasingly being recognized 110, 111. Secretion of HIF‐1*α* in response to tumor microenvironment hypoxia promotes the mesenchymal switch in TNBC cells via Hh signaling activation 95. GLI1 appears to be the mediator of HIF‐1*α*‐Hh crosstalk and hypoxia‐induced EMT. It is unclear, though, whether increased GLI1 expression in response to hypoxia involves direct regulation of GLI1 expression by HIF‐1*α* downstream of SMO, as was reported in other cancers 93, or activation of other signaling factors, such as NF‐kB, known to regulate both HIF‐1*α* 112 and GLI1 expression in TNBC cells. The extracellular matrix protein OPN also modulates the epithelial‐mesenchymal plasticity in TNBC cells by activating the Hh pathway downstream of SMO. In this respect, inactivation of GSK3*β* by OPN was shown to enhance the nuclear translocation and transcriptional activity of GLI1 55. Taking into consideration that OPN is a known GLI1 transcriptional target 113, a potential vicious cycle can be envisaged, amplifying OPN‐mediated heterotypic signaling and expansion of stem cell‐like tumor subpopulation in TNBC.

Peiris‐Pagès et al.^114^ recently proposed a model whereby exposure to cytotoxic drugs—doxorubicin, paclitaxel, and cisplatin, among others—induces metabolic and phenotypic transformation of stromal fibroblasts leading to the emergence of a highly glycolytic, autophagic, and proinflammatory microenvironment. This catabolic microenvironment, in turn, was shown to promote activation of stemness pathways, including Hh signaling, in neighboring ER+ breast cancer cells 114. Although this remains unsubstantiated, similar stromal signaling may conceivably partake in Hh signaling activation in TNBC cells following chemotherapy. Release of inflammatory cytokine after treatment with docetaxel was found to correlate with Hh signaling activation in a TNBC xenograft model of residual disease; thus, providing indirect evidence that inflammatory signaling induced by chemotherapy likely promote deregulated Hh signaling in tumor cells 115. The exact mechanisms by which stromal‐derived inflammatory signals lead to Hh signaling activation in tumor cells and their ligand‐ (and SMO‐) dependence remain to be determined.

#### Paracrine Hedgehog signaling

Paracrine Hh signaling has been implicated in stromal‐dependent tumor growth in breast cancer 56, 116 and a paracrine signature of high epithelial Hh ligand and high stromal GLI1 expression was found to be an independent predictor of decreased overall survival 56. Preclinical data also lend support to the existence of similar paracrine signaling mechanisms in TNBC 56, 117, 118.

Hh ligand secreted by TNBC cells was shown to activate paracrine Hh signaling in neighboring bone marrow stromal cells promoting their release of pro‐tumorigenic cytokines 117 and in osteoclasts precursors, thereby contributing to osteoclastogenesis and osteolysis 118. In osteoclasts specifically, breast cancer‐initiated paracrine Hh signaling is a critical event driving OPN and OPN‐induced tissue protease (MMP9 and cathepsin K) expression essential for their resorptive activity 118. Similarly, *in vivo* studies in murine models of basal‐like TNBC also demonstrate stromal‐specific Hh target gene expression driven by tumor‐secreted ligand contributing to tumor growth, invasion, and metastasis 56.

## Targeting the Hedgehog Pathway in Triple‐Negative Breast Cancer

Hh‐targeted therapies currently in use, and those that have been or are presently the subject of clinical investigation, consist of inhibitors directed against the most “druggable” pathway target, SMO (GDC‐0449, BMS‐833923, LDE‐225, PF‐04449913, LY2940680, LEQ506, and IPI‐926). Although clinical benefit from SMO inhibitors has been established in basal cell carcinoma (BCC) 119, 120 and medulloblastoma 121, their use in other solid tumors including colorectal, pancreatic, or lung cancer has been disappointing 122, 123, 124. Likewise, itraconazole, a potent SMO inhibitor 125, has also yielded modest clinical benefit when used as single agent in metastatic castrate‐resistant prostate cancer 126.

Failure of SMO inhibitors in these malignancies can be at least partly explained in that aberrant Hh signaling in these cancers, unlike medulloblastoma or BCC, is not driven by mutational mechanisms effectively targeted by SMO inhibition. Instead, deregulation of Hh signaling in these malignancies, not unlike breast cancer, is likely due to multiple coexisting mechanisms of pathway activation comprising SMO‐dependent autocrine and/or paracrine signaling and SMO‐independent signaling through direct regulation of GLI expression or activity (Table 2). Likewise, established and potential mechanisms of Hh pathway activation downstream of SMO, such as loss of tumor suppressor genes, Teashirt zinc finger homeobox 2 (TSHZ2), Liver kinase B1 (LKB1), or Singleminded‐2s (SIM2s) 127, 128, 129, provide reason to speculate that these drugs may have limited application in TNBC.

**Table 2:** Mechanisms of deregulated Hh signaling in solid tumors

Hh‐dependent tumors	
BCC	Loss of function PTCH mutation (90% of sporadic BCCs) or activating SMO mutation (10% sporadic BCC)
Medulloblastoma	Loss of function PTCH mutation (10‐20% of sporadic medulloblastoma), less commonly: activating SMO mutations or GLI amplification
Non‐Hh‐dependent tumors (Hh signaling is nonetheless implicated in tumor cell proliferation, EMT, invasion, migration and drug resistance)	
Non‐small‐cell lung cancer	Autocrine SMO‐dependent signaling: Loss of HHIP or increased HH ligand expression via SOX2‐mediated regulation of hedgehog acetyltransferase HHAT expression^a^)Ligand (SMO)‐independent signaling: Direct GLI2 activation by FGFR1 through MAPK signaling (squamous cell lung cancer)Likely non‐canonical SMO‐dependent signaling (cytoskeletal rearrangement in lung cancer cells) Paracrine stromal signaling (lung fibroblasts)
Gastric cancer	Autocrine SMO‐dependent signaling: Increased Hh ligand expression mediated by epigenetic mechanisms or NF‐kB signaling Ligand (SMO)‐independent signaling: Direct GLI1 activation by MAPK signalingParacrine stromal signaling (in stroma surrounding pseudopyloric metaplastic lesions, and fibroblasts in diffuse‐type gastric cancer)
Colorectal cancer	Autocrine SMO‐dependent signalingLigand (SMO)‐independent signaling: Direct GLI1 activation by MAPK, PI3K, and Wnt/*β*‐catenin signaling. Loss of p53 and PTEN also shown to increase GLI1 activityParacrine stromal signaling (in colorectal adenoma and invasive carcinoma)
Pancreatic cancer	Autocrine SMO‐dependent signalingLigand (SMO)‐independent signaling: Oncogenic K‐RAS induces GLI1 activity via MAPK signaling. TGF‐*β*/SMAD signaling directly activates GLI2Paracrine stromal signaling (linked to desmoplasia)
Prostate cancer	Autocrine SMO‐dependent signalingLigand (SMO)‐independent signaling: Direct GLI1 activation by RAS/MAPK and PI3K/AKT signalingParacrine stromal signaling

a Hedgehog acetyltransferase catalyzes the rate‐limiting step in Hh ligand production. Increased expression of HHAT mediated by SOX2 leads to increased Hh ligand production and ligand‐dependent autocrine signaling in squamous cell lung cancer. FGFR1: Fibroblast growth factor receptor 1.

Few SMO inhibitor trials on solid tumors have allowed enrollment of breast cancer patients and only three studies (NCT01071564, NCT01757327, and NCT02027376) investigated the use of SMO inhibitors, alone or in combination, exclusively in breast cancer. Pending formal presentation of all study results, preliminary findings suggest limited therapeutic efficacy, and with early closure of two of the breast cancer trials, it is unlikely that we will gain further insight into the clinical value of SMO inhibitors in TNBC (Table 3). In the absence of clinical data, collective findings of preclinical studies are supportive of resistance to SMO antagonists in TNBC and suggest that effective Hh pathway inhibition will necessitate the use of a GLI‐targeted approach 42, 43, 54, 55, 90.

**Table 3:** Clinical trials with SMO inhibitors allowing enrollment (ongoing studies) or with enrolled (closed studies) breast cancer patients

Hedgehog antagonists	Trial	Patient population	Combination/Comparator arm	Trial Description
SMO antagonists				
GDC‐0449 (Vismodegib)	NCT01071564Phase I (terminated)	Locally advanced unresectable or metastatic HER‐2 negative breast cancerPlanned expansion phase limited to TNBC	In combination with Notch inhibitor R04929097	Primary outcome: SafetySecondary outcomes:PK and PG dataTumor response (RECIST)Hh and BCSC marker expression
LDE‐225 (Erismodegib/Sonidegib)	NCT01576666Phase Ib	Metastatic solid tumors including breast cancer (*n* = 3)	In combination with Pan‐PI3K inhibitor buparlisib (BKM120)	Primary outcome:SafetySecondary outcomes:ORR, EPRPK data
	NCT02027376Phase Ib	Advanced and metastatic triple‐negative breast cancer (≤3 prior chemotherapy regimens for advanced/metastatic disease)	In combination with docetaxel (every 3 weeks)	Primary outcome:SafetySecondary outcomes:ORRTTPPK data
	NCT01757327Phase II (withdrawn)	Stage II and III triple‐negative breast cancer after neoadjuvant/adjuvant chemotherapy and surgery	Placebo	Primary outcome:Proportion of patients who are bone marrow disseminated tumor cell (DTC)‐negative after therapySecondary outcomes:DFS, OSPTCH1 expression
	NCT00880308Phase I 148	Advanced or metastatic solid tumors including breast cancer (*n* = 3)		Primary outcome:SafetySecondary outcomes:Tumor response (RECIST)Best response (not reported in breast cancer patients enrolled in the study) PK and PD data
LY2940680 (Taladegib)	NCT01226485NCT01919398Phase I	Advanced solid tumors		Primary outcome:SafetySecondary outcomes:Number/proportion of patients with tumor responsePK data
TAK‐441	NCT01204073Phase I [149]	Advanced solid tumors including breast cancer (*n* = 1)		Primary outcome:SafetySecondary outcomes:Tumor response (PD as best response in breast cancer patient)
LEQ506	NCT01106508Phase I	Advanced or metastatic solid tumors, medulloblastoma, BCC		Primary outcome:SafetySecondary outcomes:Tumor responsePK and PD data

BCC, Basal cell carcinoma; PK, pharmacokinetic; PG, pharmacogenetic; PD, pharmacodynamic; ORR, overall response rate; EPR, early progression rate; TTP, Time to progression; DFS, Disease‐free survival; OS, Overall survival.

So far, several GLI inhibitors have been identified but their use is currently limited to preclinical investigation. These include direct inhibitors such as GANT61, GANT58, Glabrescione B (GlaB), hedgehog pathway inhibitors (HPI) 1‐4, arsenic trioxide (ATO) and indirect inhibitors, arcyriaflavin C, physalin B/F, staurosporinone, pyrvinium, selective HDAC1/2 inhibitors, I‐BET151, and JQ1.

### Direct GLI inhibitors

These drugs directly bind to and antagonize GLI transcription factors by interfering with their processing, ciliary trafficking, or DNA binding. GANT58, GANT61, and Glabrescione B (GLaB) are thought to inhibit the transcriptional output of Hh signaling by interfering with GLI DNA‐binding 130, 131, 132, whereas HPIs exert their activity through several mechanisms including inhibition of GLI processing (HPI 1), impaired conversion of GLI to GLI_A_ (HPI 2,3) and disruption of ciliary processes required for GLI function (HPI 4) 133. Drug repurposing strategies have also identified ATO as a potent selective GLI inhibitor with two proposed mechanisms of actions: direct inhibition of the transcriptional activity of GLI1 or interference with ciliary trafficking (early effect) and enhanced degradation of GLI2 (delayed effect) 134, 135.

### Indirect GLI inhibitors

Drugs in this category target GLI proteins indirectly by interfering with mechanisms affecting their transcriptional activity, including post‐translational modifications leading to GLI activation/degradation, and epigenetic regulation. For instance, forskolin and pyrvinium enhance PKA and CK1*α*‐mediated GLI1/2 degradation, respectively, 136, 137 whereas staurosporinone and physalins B/F are thought to inhibit PKC‐*δ*/MAPK‐mediated GLI activation 138. Similarly, selective HDAC1/2 inhibitors were shown to antagonize GLI1 and GLI2 activity by counteracting their transcriptional activation induced by deacetylation 60. Epigenetic silencing of GLI1 and GLI2 proteins by JQ1 and I‐BET151 has also been described. Both drugs exert their activity by interfering with bromo and extra C‐terminal (BET) bromodomain protein 4 activity, a key activator of *GLI* transcription 139, 140.

Of all GLI inhibitors, only GANT58 and GANT61 have been evaluated in TNBC cells with more compelling data on the activity of the latter drug 42, 55, 90, 141. Despite being the most promising GLI inhibitor to date, it is uncertain whether GANT61 will be suitable for drug development given the lack of information on its pharmacokinetic profile. An important consideration to take into account for the development and selection of direct GLI inhibitors in TNBC is the existence of post‐translational processing mechanisms leading to truncated GLI isoforms which may potentially alter the binding affinity or the activity of any specific candidate drug 105.

Indirect GLI inhibitors have emerged as attractive Hh targets mostly due to their favorable characteristics for druggability compared to GLI transcription factors per se, however, their Hh inhibitory activity has not been investigated in TNBC. Taking into account the existence of several distinct oncogenic signals leading to GLI activation in TNBC and the uncertainty that extrinsic GLI activation is subject to similar regulatory mechanisms as endogenous GLI, the efficacy of indirect inhibitors remains unclear.

Furthermore, the functional redundancy of GLI proteins and the implication of both GLI1 and GLI2 in the pathogenesis of TNBC may limit the application of any direct or indirect inhibitor exhibiting preferential activity against one or the other but not both GLI proteins. Therefore, demonstration of potent GLI1/GLI2 inhibitory activity at cytotoxic concentrations will be an important criterion when selecting drugs for clinical development in TNBC.

## Conclusion and Perspectives

In this review, we have discussed the current evidence and recent studies highlighting the role of the Hh pathway in the pathogenesis of TNBC and explored mechanisms of deregulated Hh signaling in this breast cancer subtype. The key contribution of extrinsic oncogenic signals, mediated by TGF‐*β*, RAS/MAPK signaling, OPN, NF‐kB, FOXC1, and HIF‐1*α*, in driving Hh pathway activation downstream of SMO in TNBC was also reviewed.

Although we have limited information on the use of SMO inhibitors in breast cancer in the clinical setting, the preclinical data we presented suggest resistance to SMO inhibitors in TNBC and instead provide the rationale for a GLI‐targeted approach.

The contribution of GLI activation in circumventing SMO inhibition in BCC or medulloblastoma and the recognition of the role of direct mechanisms of GLI activation downstream of SMO in drug resistance, tumor growth, and progression in other solid tumors have provided impetus for identifying potent GLI inhibitors. So far, several direct and indirect inhibitors of GLI have been discovered; yet, their suitability for clinical use is still uncertain. Based on currently available data, the use of direct inhibitors with GLI1/GLI2 activity such as GANT61 may represent the most effective approach at targeting Hh signaling in TNBC. Determining whether epigenetic and post‐translational regulatory mechanisms targeted by indirect GLI inhibitors can be overcome by extrinsic GLI activation will be important to ascertain the potential activity of these drugs in TNBC.

Additional questions warranting further investigation include:

*Which TNBC patients might benefit from Hh‐targeted interventions?*Hh pathway deregulation may not necessarily play a pathogenic role in all subtypes of TNBC; hence, Hh inhibition may prove to be of limited value among unselected patients. A recent analysis of a dataset of over 330 tumor samples yielded evidence for preferential GLI1 expression in claudin‐low relative to other basal tumors and higher sensitivity to GLI inhibition was observed in claudin‐low cells lines. Recognizing the heterogeneity of TNBC and limiting future studies to specific molecular subtypes, such as claudin‐low subtype, enriched for BCSCs may potentially identify subgroups of TNBC patients who might benefit from Hh‐targeted interventions.In addition, no biomarker predictive of clinical benefit from SMO antagonists in other solid tumors has been identified to date. It is uncertain as to whether or not this endeavor has been unsuccessful due to ineffective Hh signaling inhibition in these cancers, which, not unlike TNBC, are characterized by downstream pathway activation not targeted by SMO antagonists. As the development of GLI inhibitors moves forward, integration of putative predictive biomarkers in clinical studies evaluating the use of these drugs in TNBC will be of paramount importance.
*When should Hh‐targeted intervention be timed during treatment?*Data presented in this review implicate chemotherapy‐induced Hh signaling activation in BCSC expansion and tumor regrowth in TNBC. It is unclear whether chemotherapy “selects” for the survival of BCSCs subpopulations in TNBC or promotes stromal metabolic stress leading to the acquisition of stem‐like phenotype by differentiated tumor cells. Nevertheless, based on the current evidence, targeting Hh signaling after completion of chemotherapy seems intuitive and the application of Hh‐targeted strategies in the future may conceivably be limited to TNBC patients who have failed to achieve clinical or pathologic complete response to neoadjuvant chemotherapy. Studies evaluating mechanisms of deregulated Hh signaling following chemotherapy in TNBC are scarce. In the absence of these data, one cannot assume that these mechanisms are identical to those driving Hh signaling in chemotherapy‐naïve TNBC. Further insight into the signaling machinery contributing to deregulated Hh signaling following chemotherapy in TNBC, both in preclinical models of residual tumor and clinical tumor samples after neoadjuvant chemotherapy, is therefore necessary.
*Should drug combination strategies be used when planning Hh‐targeted therapeutic interventions in TNBC?*Hh, TGF‐*β*, PI3K/AKT, RAS/MAPK signaling possess overlapping functionality in TNBC; potential crosstalk between Hh and these signaling pathways could potentially circumvent pharmacological Hh inhibition. Thus, uncovering mechanisms of crosstalk between Hh signaling and other key tumorigenic pathways in TNBC can provide the basis for the development of effective combination therapies. A synergistic therapeutic effect has been reported in non‐mammary solid tumors when combined targeting of GLI and PI3K/AKT or GLI and EGFR signaling was used 142, 143, 144, 145. Exploring potential synergy between Hh and EGFR signaling specifically may be of particular relevance in TNBC, as the rationale for EGFR inhibitor failure in this breast cancer subtype remains elusive.
*Is there a role for stromal Hh signaling inhibition in TNBC?*Despite the potential resistance of TNBC cells to SMO modulation, stromal paracrine signaling is, on the other hand, SMO‐dependent and consequently may be amenable to modulation by SMO antagonists. Classically, Hh signaling in the stromal microenvironment was thought to foster tumor growth and invasion, and corroborative preclinical data have revealed potential benefit for the use of SMO modulation and stromal inhibition in TNBC 117, 118. However, recent reports have challenged the hypothesis of pro‐tumorigenic stromal Hh signaling demonstrating, contrariwise, that stromal signaling may exert a tumor suppressive and antiangiogenic role in pancreatic cancer 146, 147. Based on these findings, Hh inhibition and stromal depletion might seemingly be a counterintuitive approach for preventing tumor progression. Extrapolation of similar conclusions in TNBC is difficult given the differences between oncogenic mechanisms driving tumor and stromal Hh signaling in pancreatic cancer and TNBC. Accordingly, further investigation is needed to better understand the mechanisms of stromal Hh signaling in TNBC and the potential effect of stromal modulation on tumor growth both in early and late‐stage disease.

## Conflict of Interest

The authors declare they have no competing interests.
